# Editorial: Intracranial aneurysms, AVM and other vascular malformations, and connective tissue disorders as potential causes of stroke: advances in diagnosis and therapeutics including novel neurosurgical techniques

**DOI:** 10.3389/fneur.2025.1661477

**Published:** 2025-07-31

**Authors:** Vishal Chavda, Giuseppe Emmanuele Umana, Bipin Chaurasia, Stefano Maria Priola, Luis Rafael Moscote-Salazar

**Affiliations:** 1 Department of Medicine and Critical Care, Multispeciality, Trauma and ICCU Center, Sardar Hospital, Ahmadabad, Gujarat, India; 2 Department of Neurosurgery, KORE University, Enna, Italy; 3 Department of Neurosurgery, College of Medical Sciences, Birgunj, Nepal; 4 Department of Neurosurgery, Northern Ontario School of Medicine University, Sudbury, Canada; 5 Research Department, AV Healthcare Innovators, LLC, Madison, WI, United States; 6 Columbian Clinical Research Group in Neurocritical Care, Bogota, Columbia

**Keywords:** intracranial aneurysm, arteriovenous malformation (AVM), stroke prevention, hemodynamic modeling, connective tissue disorders, endovascular neurosurgery

The cerebral vasculature is a marvel of complexity and when disrupted by aneurysms, AVMs, or vascular malformations associated with connective tissue disorders, it becomes a critical substrate for ischemic and hemorrhagic stroke. This Research Topic in *Frontiers in Neurology* curates multidimensional contributions that unify genetics, computational modeling, clinical diagnostics, and procedural advances, collectively moving the field toward more individualized and proactive care models.

Systemic inflammation and autoimmune disease have emerged as independent risk factors for aneurysm formation. Tang et al. employed Mendelian randomization to demonstrate a causal link between systemic lupus erythematosus and intracranial aneurysms, with consistent findings across East Asian and European populations, advocating for targeted vascular imaging in autoimmune patients (Tang et al.). Complementing this, Wang et al. integrated high-resolution vessel wall imaging with hemodynamic and clinical features to produce a validated rupture risk model (Wang et al.), while Lei et al. applied machine learning to predict poor outcomes after endovascular treatment highlighting the growing utility of AI-based clinical decision support (Lei et al.). Sex- and age-specific risk differentials were explored by Mao et al. and Shen et al., both showing that anatomical and physiological differences particularly in women under 50 and men with certain aneurysm morphologies necessitate tailored therapeutic strategies (Mao et al.; Shen et al.).

Beyond etiology, the procedural landscape continues to evolve. Zhang et al. reported that stent-assisted recanalization in chronic occlusions can be safe and effective when proper anatomical factors are respected (Zhang et al.). Meanwhile, Lan et al. refined the subtemporal approach to access posterior communicating artery aneurysms, demonstrating it as a viable alternative in anatomically challenging cases (Lan et al.). Hemodynamic modeling, particularly computational fluid dynamics (CFD), is increasingly foundational. Xu et al. utilized patient-specific CFD to shape microcatheters in aneurysm embolization, improving precision and procedural safety (1), while Bozorgpour and Kim emphasized the need for standardized hemodynamic variables in CFD analysis, proposing a framework for cross-study reproducibility (2).

In AVMs, bibliometric analysis by Tang et al. revealed a 20-year trend toward combined modality treatments and an increased focus on outcome prediction, especially in unruptured cases (3). Shotar et al. contributed a unique dataset on ultra-early neurological deterioration after AVM rupture, underscoring the need for rapid diagnosis and decompression protocols (Shotar et al.). Genetic investigations by Neyazi et al. found that CEACAM1 and IL-6 polymorphisms, along with sex-based immunologic differences, may underlie hemorrhagic risk in AVM carriers, suggesting that inflammation is both a symptom and a pathogenic driver (4). Finally, the issue turns a spotlight on connective tissue disorders where under-recognized risk meets preventable catastrophe. Kim et al. reported aneurysm prevalence as high as 28% in patients with Ehlers-Danlos, Marfan, and Loeys-Dietz syndromes far exceeding the general population rate of 3% (5). These data argue for systematic screening in patients with heritable vasculopathies and call for collaboration between neurologists, geneticists, and vascular surgeons.

In summary, this Research Topic crystallizes three pillars of modern vascular neurology: inflammation and genetics as risk predictors, hemodynamic modeling as both diagnostic and therapeutic aid, and procedural refinement as a vehicle for safer, personalized interventions. Despite these gains, several gaps persist particularly in unifying risk scores, validating CFD parameters across platforms, and embedding genomic screening into standard practice. The future of stroke prevention in vascular malformations lies not in a single discipline but in cross-disciplinary synthesis, where predictive algorithms, surgical precision, and molecular insight converge at the bedside.
