# Correction: Editorial: Intracranial aneurysms, AVM and other vascular malformations, and connective tissue disorders as potential causes of stroke: advances in diagnosis and therapeutics including novel neurosurgical techniques

**DOI:** 10.3389/fneur.2025.1750900

**Published:** 2026-03-12

**Authors:** Vishal Chavda, Giuseppe Emmanuele Umana, Bipin Chaurasia, Stefano Maria Priola, Luis Rafael Moscote-Salazar

**Affiliations:** 1 Department of Medicine and Critical Care, Multispeciality, Trauma and ICCU Center, Sardar Hospital, Ahmadabad, Gujarat, India; 2 Department of Neurosurgery, KORE University, Enna, Italy; 3 Department of Neurosurgery, College of Medical Sciences, Birgunj, Nepal; 4 Department of Neurosurgery, Northern Ontario School of Medicine University, Sudbury, Canada; 5 Research Department, AV Healthcare Innovators, LLC, Madison, WI, United States; 6 Columbian Clinical Research Group in Neurocritical Care, Bogota, Columbia

**Keywords:** intracranial aneurysm, arteriovenous malformation (AVM), stroke prevention, hemodynamic modeling, connective tissue disorders, endovascular neurosurgery

In the published article, several errors were identified in the reference list:

1. Xu Q, Wang R, Wang M, Liu Z, Zhang P. Hemodynamic modelingassisted microcatheter shaping improves outcomes in endovascular treatment of posterior communicating artery aneurysms. *Front Neurol*. (2024) 15:1406531. doi: 10.3389/fneur.2024.1406531

2. Bozorgpour A, Kim ST. A comprehensive review of hemodynamic parameters in computational fluid dynamics studies of intracranial aneurysms. *Front Neurol*. (2024) 15:1390768. doi: 10.3389/fneur.2024.1390768

3. Tang J, Wu W, Chen Z, Luo J, Fang Z. Research trends in arteriovenous malformations over the past 20 years: a bibliometric analysis. *Front Neurol*. (2024) 15:1327915. doi: 10.3389/fneur.2023.1327915

4. Neyazi B, Veldeman M, Vychopen M, Hanggi D, Zhang J, Sako W, et al. Sex differences and inflammatory biomarkers in brain arteriovenous malformations: a clinical-genomic correlation. *Front Neurol*. (2024) 15:1391397. doi: 10.3389/fneur.2024.1391397

5. Kim ST, Brinjikji W, Kallmes DF. Prevalence of intracranial aneurysms in patients with connective tissue diseases: a retrospective study. *AJNR Am J Neuroradiol*. (2016) 37:1422–6. doi: 10.3174/ajnr.A4718

The corrected references appear below:

1. Xu G, Ba Y, Zhang K, Cai D, Yang B, Zhao T, et al. Application of microcatheter shaping based on computational fluid dynamics simulation of cerebral blood flow in the intervention of posterior communicating aneurysm of the internal carotid artery. *Front Neurol*. (2023) 14:1221686. doi: 10.3389/fneur.2023.1221686

2. Bozorgpour R, Kim P. CFD-based quantification of hemodynamic variables in cerebral aneurysms: how hemodynamics shape aneurysm fate. *arxiv* [*Preprint*]. (2025). arXiv:2505.14695. doi: 10.48550/arXiv.2505.14695

3. Tang W, Chen Y, Ma L, Chen Y, Yang B, Li R, et al. Current perspectives and trends in the treatment of brain arteriovenous malformations: a review and bibliometric analysis. *Front. Neurol*. (2024) 14:1327915. doi: 10.3389/fneur.2023.1327915

4. Neyazi B, Herz A, Stein KP, Gawish I, Hartmann C, Wilkens L, et al. Brain arteriovenous malformations: implications of CEACAM1-positive inflammatory cells and sex on hemorrhage. *Neurosurg Rev*. (2017) 40:129–34. doi: 10.1007/s10143-016-0744-5

5. Kim ST, Brinjikji W, Kallmes DF. Prevalence of intracranial aneurysms in patients with connective tissue diseases: a retrospective study. *Am J Neuroradiol*. (2016) 37:1422–6. doi: 10.3174/ajnr.A4718

A correction has also been made to the Generative AI statement. The original statement read: “The author(s) declare that no Gen AI was used in the creation of this manuscript”. This has been corrected to:

The author(s) declared that generative AI was used to correct the grammar and language of this manuscript.

The original version of this article has been updated.

